# Supplementary material for: The Contribution of Psychological Factors to Inter-Individual Variability in Conditioned Pain Modulation Is Limited in Young Healthy Subjects
Source: Brain Sci. 2022 May 10;12(5):623. doi: 10.3390/brainsci12050623 (PMC9139004; doi:10.3390/brainsci12050623)
Supplement: Supplementary file 1 [file brainsci-12-00623-s001.zip › brainsci-1665233-supplementary.pdf]

Supplementary Materials

Table S1. Cross-sectional analysis including interactions between psychological factors and CPM paradigm (linear regression, Model 4, *n* = 126) for psychological factor interaction with paradigm. Model 4: multiple R^2 = 4.6%, *p* = 0.845. Interaction effects between BDI, STAI Trait, or PCS and paradigm are signified with '*'. Sex and paradigms compared to a reference (male and 30 s heat/60 s cold, respectively). Paradigm 1 = heat 60 s/cold 90 s, Paradigm 2 = electrical/cold120s. CS_{Temp} = conditioning stimulus temperature in °C, BDI = Beck's Depression Inventory score, STAI Trait = State Trait Anxiety Inventory score (trait subscale), PCS = Pain Catastrophizing Scale score.

Model	Predictor	Estimate	*p*-Value
Model 4	CS_{Temp}	1.15	0.167
	Age	−0.15	0.694
	Sex	1.31	0.826
	BDI	−0.05	0.961
	PCS	−0.53	0.190
	STAI Trait	−0.35	0.485
	Paradigm 1	−5.73	0.862
	Paradigm 2	−27.2	0.351
	BDI*Paradigm 1	0.51	0.827
	BDI*Paradigm 2	−0.10	0.975
	STAI Trait*Paradigm 1	0.03	0.978
	STAI Trait*Paradigm 2	0.70	0.444
	PCS*Paradigm 1	0.41	0.545
	PCS*Paradigm 2	0.54	0.498

Table S2. Repeated measures analysis including interactions between psychological factors and CPM paradigm (mixed model analysis, Model 5, 52 participations, 118 observations). REML criteria at convergence = 1006.3. Interaction effects between psychological factors and paradigm are signified with '*'. Sex and paradigm compared to a reference (male and electrical/120 s cold, respectively). CS_{Temp} = conditioning stimulus temperature in °C, BDI = Beck's Depression Inventory score, STAI Trait = State-Trait Anxiety Inventory score (trait subscale), PCS = Pain Catastrophizing Scale score. Significant effects are marked in bold.

Model	Predictor	Estimate	*p*-Value
model 5	**CS_{Temp}**	**1.56**	**0.002**
	Age	0.29	0.541
	Sex	−4.30	0.445
	BDI	2.76	0.686
	PCS	−0.16	0.943
	STAI Trait	−0.31	0.634
	Paradigm	−7.26	0.198
	Repeat	−2.95	0.209
	BDI*Paradigm	−3.76	0.130
	STAI Trait*Paradigm	0.22	0.795
	PCS*Paradigm	0.24	0.709

Table S3. Significance and variance explained by fixed effects in Models 6 and 7 (n = 52, 118 observations). Interaction effects are denoted by '*'. Model 6 R^2: 10.0%, Model 7 R^2: 13.6%. p-values signify the significance of the predictor, not the significance of explained variance. Please note that the combined variances of Models 6 or 7 differ from the combined fixed effects variances of Models 3 and 5, respectively, due to different model types (linear vs. mixed) and different algorithms used in variance decomposition. CS_{Temp} = conditioning stimulus temperature in °C, BDI = Beck's Depression Inventory score, STAI Trait = State-Trait Anxiety Inventory score (trait subscale), PCS = Pain Catastrophizing Scale score.

Predictor	Model 6		Model 7	
	Variance Explained	*p*-Values	Variance Explained	*p*-Value
CS_{Temp}	4.55%	0.024	4.50%	0.022
Age	0.06%	0.659	0.06%	0.494
Sex	0.01%	0.903	0.02%	0.523
BDI	0.08%	0.584	0.08%	0.116
STAI Trait	0.09%	0.920	0.09%	0.413
PCS	<0.01%	0.983	<0.01%	0.626
Paradigm	2.07%	0.151	2.11%	0.728
Repeat	0.09%	0.212	0.10%	0.242
BDI*Paradigm	n/a	n/a	2.95%	0.053
PCS*Paradigm	n/a	n/a	0.02%	0.561
STAI Trait*Paradigm	n/a	n/a	0.04%	0.708

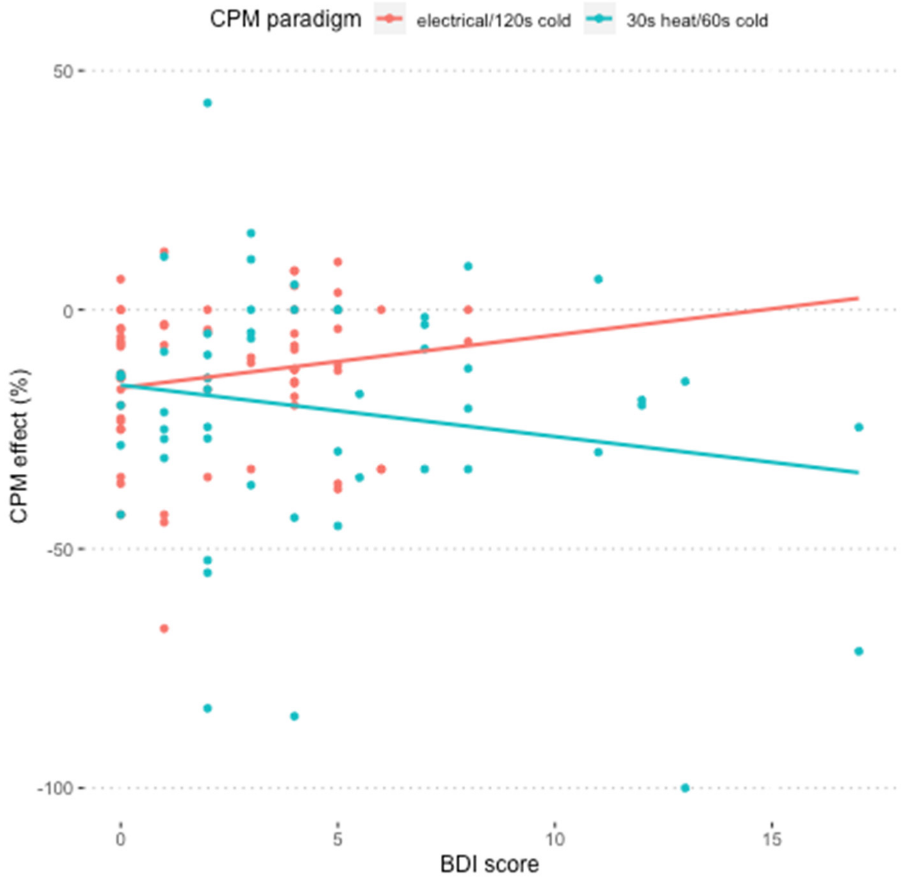

Figure S1. Interaction plot of the effect of BDI scores on CPM magnitude in CPM paradigms using electrical and heat stimulation as test stimuli, respectively.

A more negative CPM effect denotes a larger pain reduction. The correlation between BDI score and CPM effects was: 0.16 and −0.18 for electrical and heat paradigms, respectively.
